# Evaluation of pre-Omicron population immunity for SARS-CoV-2 in the Household Influenza Vaccine Evaluation (HIVE) cohort

**DOI:** 10.1371/journal.pone.0360506

**Published:** 2026-10-15

**Authors:** Yangyupei Yang, Casey Juntila-Raymond, Amy Callear, Matthew Smith, Reilly K. Atkinson, Yan Zhao, Ruhi Shah, Jefferson J. S. Santos, Scott E. Hensley, Arnold S. Monto, Emily T. Martin

**Affiliations:** 1 Department of Epidemiology, University of Michigan School of Public Health, Ann Arbor, Michigan, United States of America; 2 Department of Microbiology, Perelman School of Medicine, University of Pennsylvania, Philadelphia, Pennsylvania, United States of America; Central University of Tamil Nadu, INDIA

## Abstract

**Background:**

Continued serosurvey for SARS-CoV-2 population immunity is critical for vaccine strain selection and identification of susceptible groups such as young children. We aim to evaluate the use of a high-throughput, low-volume assay to describe strain-specific IgG measurement in a longitudinally followed community based cohort.

**Methods:**

The longitudinal Household Influenza Vaccine Evaluation study has observed around 300 households annually for acute respiratory illness. SARS-CoV-2 variant-specific IgG (including original, Delta, Beta, and Omicron variants) concentrations were measured using multiplex electrochemiluminescence (ECL), pseudoviral neutralization (PN), and ACE2 inhibition assays in participants who had serum samples drawn between July 1 and December 31, 2021. Spearman’s rank correlation coefficient assessed correlation within and between ECL, ACE2, and PN assays. Receiver operating characteristic (ROC) analysis evaluated the performance of ECL and ACE2 assays. Generalized additive mixed models estimated population immunity by age against SARS-CoV-2 variants.

**Results:**

A total of 156 serum samples from 117 participants were selected for this study. Most (65.8%) were from adults and a majority (77.8%) received vaccination during the analysis period. High correlations were observed within ECL IgG assays (0.95–1.00), within ACE2 inhibition assays (0.70–0.99), and between assay platforms, including ECL versus PN (0.81–0.83) and ACE2 versus PN (0.88–0.95). ROC analysis demonstrated strong discrimination of vaccination status by ECL IgG assays (AUC: 0.84–0.88; sensitivity: 0.88–0.91; specificity: 0.87–0.88). ACE2 inhibition assays showed more variable performance (AUC: 0.53–0.96; sensitivity: 0.17–0.93; specificity: 0.91–1.00), with lower performance observed for some BA.2-related antigens. SARS-CoV-2 IgG concentrations generally increased with age and appeared to plateau in early adulthood, although estimates were imprecise in younger age groups because of small sample sizes.

**Conclusion:**

We found a high correlation between ECL, ACE2, and PN across variants. Age-specific IgG quantities reflected behavior and infection histories collected by active surveillance.

## Introduction

COVID-19, caused by the SARS-CoV-2 virus, has shown a significant health burden globally, characterized by its rapid evolution and severe disease outcomes [1]. The immunoglobulin G (IgG) generated either post-vaccination or through natural infection is pivotal in preventing severe outcomes and, to some extent, subsequent infections [2]. Cross protection has been observed where IgG responses to one strain may cross protect against other strains [3]. Serological assays, which quantify specific immunoglobulins such as IgG neutralizing or functional antibodies, are important for understanding population immunity. Pseudovirus neutralization (PN) assays, a functional test that requires blocking viral entry and replication, typically focus on a single protein target per test and are technically challenging [4]. The complexity of immune responses to fast-evolving SARS-CoV-2 strains needs a broader approach. Multiplex electrochemiluminescence (ECL) assays assess antibody binding and can simultaneously detect multiple antigens. However, concerns remain that binding assays may not adequately assess the capacity to block infection and could potentially lack specificity against different variants. Another competition multiplex assay evaluates antibodies’ capacity to inhibit the binding of the angiotensin-converting enzyme 2 (ACE2) to SARS-CoV-2 antigen, thus analyzing antibody activity through ACE2 binding inhibition. Multiplex assays provide a robust and scalable solution for comprehensive immune profiling that can be used for large population-based serosuveys [5,6].

We conducted a multi-age community serosurvey of pre-Omicron antibody levels using a multiplex coronavirus immunoassay from Meso Scale Diagnostics (MSD, Rockville, USA) that utilizes multiplex electrochemiluminescence assay for the detection of antibodies against various protein targets, including the Spike protein, the Receptor Binding Domain (RBD) of the spike protein, and the nucleocapsid protein (N) of several SARS-CoV-2 strains. Additionally, our study used the ACE2 multiplex assay for antibodies that block the binding of ACE2 to Spike antigens from variants of SARS-CoV-2 to assess binding inhibition against various SARS-CoV-2 antigens. These assays were compared to PN tests, providing a comprehensive evaluation of the baseline antibody profile of participants in the longitudinal Household Influenza Vaccine Evaluation (HIVE) cohort conducted in Michigan before the spread of the Omicron variant. A major strength of the longitudinal HIVE cohort is the availability of repeated, high-quality immunologic measurements that capture longitudinal antibody immunity along with complete vaccination and infection history, making this a unique resource for nested serosurveys [7–12].

The period before widespread Omicron transmission (July 1^st^ – December 31^st^ 2021) offers a unique opportunity to study SARS-CoV-2 infection- and vaccine-induced immunity, as infection prevalence was low compared to the Omicron period, and vaccines had not yet been introduced to children. This created a clear distinction between infection-induced and vaccine-induced immunity, with few cases of infection in vaccinated individuals (i.e., “hybrid immunity”).

## Materials and methods

### Study population and serum samples

Recruitment and retention procedures of HIVE study have been previously described [13]. Briefly, since 2010, eligible households must have ≥3 persons living at the same address with at least one child aged <10 years at the time of initial enrollment. In mid‐2020, eligibility was expanded to those with at least one child aged <21 years. Participants contributed blood specimens at enrollment, twice annually, pre- and post-flu and COVID-19 vaccination. For this analysis, serological samples were selected among HIVE participants who provided blood specimens between July 1^st^ and December 31^st,^ 2021.

The HIVE study was approved by the University of Michigan Institutional Review Board. All participants were enrolled prospectively after completion of the informed consent process. Adults aged ≥18 years provided written informed consent for their own participation. For participants aged <18 years, written permission was obtained from a parent or legal guardian before participation. Children aged ≥7 years additionally provided verbal assent before study procedures were performed. The present analysis used serum specimens and study data collected under this approved longitudinal cohort protocol.

### Demographic information and household characteristics

Each participant, or their parent or legal guardian, completed an enrollment survey detailing demographics, household characteristics, chronic medical conditions, COVID-19 vaccination history, and prior SARS-CoV-2 infection. Participants were surveyed annually to update demographic and household information.

Weekly surveillance for COVID-19 symptoms was conducted. Households received weekly email reminders to report acute respiratory illnesses (ARIs) (see Supplementary Materials) at symptom onset (within 7 days post-onset) of qualifying ARIs by parents or through self-collection. All respiratory specimens were tested for SARS-CoV-2 using multiplex reverse transcriptase-polymerase chain reaction (RT-PCR).

Pre-Omicron Individual immunity profiles were classified into four categories: 1) Vaccination: participants who received at least the primary doses of COVID-19 vaccination as defined by the CDC; 2) Infection: participants with RT-PCR confirmed SARS-CoV-2 symptomatic infection before the emergence of Omicron; 3) Hybrid Immunity: participants who received at least the primary doses of COVID-19 vaccination and had RT-PCR confirmed SARS-CoV-2 infection before Omicron; 4) No vaccination, no infection.

### Serological assays

Serum specimens were screened for IgG antibodies to the SARS-CoV-2 spike, RBD, and N protein, using a commercially available electrochemiluminescence (ECL) immunoassay kit Panel 29 (Spike antigens: original, B.1.351, B.1.617.2, BA.2, BA.2.12.1, BA.2.75, BA.4, BA.5)) and 30 (RBD antigens: original, B.1.1.7 B.1.351, BA.2, BA.2.12.1, BA.2.75, BA.4/5, delta. A result of ≥5000AU/mL N protein was considered seropositive [14]. Serum samples were diluted 1:5000 and 1:50,000 and run in duplicate on each plate and processed following the manufacturer's protocol [15–17]. Two serology controls (SC 1.2 and SC 1.3) provided by the manufacturer, containing known concentrations of IgG antibodies against the targets in the panel, were run in duplicate on each plate. Four independent runs for each sample were used to calculate coefficient of variation (CV) for quality control. Assay data were processed using standardized R code to resolve duplicates and reflex testing to higher dilutions (supplementary material).

Competition ACE2 assays were used to evaluate the antibody inhibition capacity against SARS-CoV-2 antigens, specifically targeting spike and RBD proteins [18,19]. The percentage inhibition was calculated relative to the assay calibrator (maximum 100% inhibition) using the equation:

Inhibition=[1−(Average Sample ECL Signal/Average ECL Signal of Calibrator)]×100%.

PN assays were conducted to measure serum titers of neutralizing antibodies against Wild Type (WT) and BA.1 Spike strains, as previously described [20,21]. Briefly, VeroE6/TMPRSS2 cells were seeded at 2.5x10^4^ cells/well in collagen-coated 96-well plates overnight. The following day, heat-inactivated sera were serially diluted 2-fold and mixed with ~300 focus-forming units per well of each VSVΔG-RFP SARS-CoV-2 pseudotype virus. A mouse anti-VSV G monoclonal antibody, 1E9F9, was spiked into virus-antibody mixtures at 600 ng/mL to ensure neutralization of any VSV-G carryover virus from pseudotype virus production. Virus-antibody mixtures were incubated for 1 h at 37 °C and then transferred to VeroE6/TMPRSS2 cells. After 22 hours postinfection at 37˚C, cells were washed with PBS, fixed with 4% PFA, and then blotted dry. Fluorescent foci were visualized and counted on an ImmunoSpot S6 analyzer. The focus reduction neutralization titer 50% (FRNT50) was measured as the greatest serum dilution at which the foci count was reduced by at least 50% relative to control cells that were infected with pseudotype virus in the absence of participant serum. FRNT50 titers for each sample were measured in two replicates.

### Statistical analysis

Antibody concentrations were analyzed for individual Spike, RBD, and N antigens measured on the ECL panels, as well as PN titers against wild-type and BA.1 spike. Seropositivity for N was defined using the manufacturer-provided cutoff 5000AU/mL by ECL assay. Spearman correlation was used to compare antibody levels within and between ECL panel variants, and to compare between PN and ECL assays. Geometric mean concentration (GMC) and 95% confidence interval (95%CI) were used to assess the GMCs of antibody concentrations for each strain among age categories. A receiver operating characteristic (ROC) curve analysis was performed to evaluate the ECL and ACE2 assay performance by antigen and protein targets. One serum sample per participant was included to ensure independence of observations. When participants contributed more than one serum sample during the study period, the earliest available sample was selected. Participants in the vaccinated-only and hybrid-immunity groups were classified as vaccinated, whereas participants in the prior-infection-only and unvaccinated-and-uninfected groups were classified as unvaccinated. ROC curves were generated, and the area under the curve (AUC) was calculated to assess discrimination. The optimal classification threshold was determined using Youden’s Index, which is the point on the receiver operating curve where sensitivity plus specificity is maximal, and aims to select an optimal threshold value for a diagnostic test [22]. At this threshold, sensitivity, specificity, and their 95% confidence intervals (CIs) were calculated using the Wilson score method. A generalized additive mixed model was used to fit antibody trends by age and SARS-CoV-2 strains pre-Omicron. All analyses were conducted using R version 4.0.38 [23], and plots were generated using the ggplot2 package.

## Results

### Participants and samples

Among the participants enrolled in the HIVE cohort, 156 serum samples from 117 participants were selected for this study (Table 1). Most participants were adults aged ≥18 years (n = 77, 65.8%), while 32 (27.4%) were aged 5–17 years and 7 (6.0%) were younger than 5 years. A slightly higher proportion of participants were female (n = 69, 59.5%) than male (n = 47, 40.5%). Participants were categorized as vaccinated only (n = 71, 60.7%), hybrid immunity (n = 20, 17.1%), unvaccinated and uninfected (n = 22, 18.8%), or prior infection only (n = 4, 3.4%). Among adults aged ≥18 years, 97.4% (75/77) had received COVID-19 vaccines, including 61 vaccinated-only participants and 14 with hybrid immunity. In contrast, among participants younger than 18 years, 53.8% (21/39) were unvaccinated and uninfected and 7.7% (3/39) had prior infection only. The majority of participants were White (n = 87, 75.0%), non-Hispanic (n = 112, 96.6%), and 34.5% (n = 40) reported at least one high-risk medical condition.

**Table 1:** Demographic characteristics of HIVE participants included in the pre-Omicron serosurvey, stratified by SARS-CoV-2 immune status.

Characteristic	Overall (N = 117)^1^	Hybrid immunity (N = 20)^1,2^	Vaccinated only (N = 71)^1,3^	Unvaccinated and uninfected (N = 22)^1,4^	Prior infection only (N = 4)^1,5^	P value^6^
**Age category, years**						<0.001
<5	7 (6.0)	0 (0.0)	0 (0.0)	7 (31.8)	0 (0.0)	
5–17	32 (27.4)	6 (30.0)	9 (12.7)	14 (63.6)	3 (75.0)	
≥18	77 (65.8)	14 (70.0)	61 (85.9)	1 (4.5)	1 (25.0)	
**Sex**						0.023
Male	47 (40.5)	8 (40.0)	22 (31.4)	15 (68.2)	2 (50.0)	
Female	69 (59.5)	12 (60.0)	48 (68.6)	7 (31.8)	2 (50.0)	
**Race**						0.81
White	87 (75.0)	17 (85.0)	51 (72.9)	15 (68.2)	4 (100.0)	
Black or African American	6 (5.2)	0 (0.0)	3 (4.3)	3 (13.6)	0 (0.0)	
Asian and/or Pacific Islander	14 (12.1)	2 (10.0)	8 (11.4)	4 (18.2)	0 (0.0)	
Other	9 (7.8)	1 (5.0)	8 (11.4)	0 (0.0)	0 (0.0)	
**Ethnicity** ^**7**^						0.39
Non-Hispanic	112 (96.6)	20 (100.0)	68 (97.1)	20 (90.9)	4 (100.0)	
Hispanic	4 (3.4)	0 (0.0)	2 (2.9)	2 (9.1)	0 (0.0)	
**Education** ^**8**^						0.88
High school graduate or equivalent	1 (1.3)	0 (0.0)	1 (1.7)	0 (0.0)	0 (0.0)	
Some university	6 (7.9)	1 (7.1)	5 (8.3)	0 (0.0)	0 (0.0)	
University graduate	24 (31.6)	5 (35.7)	18 (30.0)	1 (100.0)	0 (0.0)	
Master's degree	28 (36.8)	3 (21.4)	24 (40.0)	0 (0.0)	1 (100.0)	
Advanced graduate degree (e.g., PhD, JD, MD)	17 (22.4)	5 (35.7)	12 (20.0)	0 (0.0)	0 (0.0)	
**High-risk medical condition**	40 (34.5)	6 (31.6)	32 (45.7)	2 (9.1)	0 (0.0)	0.005

^1^Values are presented as n (%).

^2^Hybrid immunity was defined as receipt of at least one dose of COVID-19 vaccine and a RT-PCR confirmed SARS-CoV-2 infection prior to Omicron emergence.

^3^Vaccinated only participants had received at least one dose of COVID-19 vaccination but had no RT-PCR confirmed SARS-CoV-2 infection prior to Omicron emergence.

^4^Prior infection only participants had RT-PCR confirmed SARS-CoV-2 infection but had not received any COVID-19 vaccination.

^5^Unvaccinated and uninfected participants had neither vaccination nor documented infection.

^6^P values were calculated using Pearson's chi-square test or Fisher's exact test, as appropriate based on expected cell counts.

^7^Race and ethnicity information was missing for one participant.

^8^Education information was collected among adult participants only and was missing for one adult participant.

### Comparison of multiplex electrochemiluminescence and pseudo-neutralization

Correlations between ECL measured IgG concentrations against Spike and RBD antigens were high across all strains, ranging from 0.954 to 0.996 (S1 Fig). Cross‑assay correlations were evaluated for matched variants only. Specifically, ECL Spike and RBD IgG concentrations were compared with PN titers for the same variant, and correlations were moderately high but lower than within‑panel ECL correlations for both Spike and RBD panels, ranging from 0.811 to 0.827. Spike and RBD ACE2 inhibition showed high correlation with PN titers for matched variants, ranging from 0.877 to 0.953. At comparable PN titer levels, ECL IgG concentrations were higher for the B.1.351 (Beta) and B.1.617.2 (Delta) variants compared to other variants.

### Antibody concentration relationship between antigens

Among 156 serum samples, 17 (10.9%) were N-positive. Of these 17 N-positive samples, 8 were collected from participants with a documented PCR-confirmed SARS-CoV-2 infection before serum collection, whereas 9 were collected from participants without a documented prior PCR-confirmed infection.

Spearman correlation coefficients were high across all antigen pairs: 0.991–0.999 (p < 0.05) for Spike IgG, 0.974–0.999 (p < 0.05) for RBD IgG, 0.866–0.992 (p < 0.05) for Spike ACE2, and 0.701–0.990 (p < 0.05) for RBD ACE2. Correlations between BA.2 and other variants were lower in ACE2 inhibition assays: 0.436–0.714 for Spike ACE2 and 0.329–0.435 for RBD ACE2 (S1 Fig). These results indicate small heterogeneity in antibody cross-reactivity across variants and assay types.

### Assay sensitivity and specificity

ROC curve analysis was conducted to evaluate the diagnostic performance of each antigen for distinguishing vaccinated from unvaccinated participants. Among IgG assays, discrimination was consistently high, with AUC values ranging from 0.84–0.88 for IgG Spike and RBD antigens (S1 Table). Optimal thresholds determined using Youden's Index yielded high sensitivity (0.88–0.91) and specificity (0.87–0.88) across all IgG antigens. ACE2 inhibition assays generally demonstrated higher specificity (0.91–1.00) and AUC values ranging from 0.53 to 0.96, although performance varied substantially across variants. For Spike antigens, AUC values ranged from 0.64 for BA.2 to 0.96 for B.1.351, while RBD antigens ranged from 0.53 for BA.2.75 to 0.95 for Alpha and Delta variants. Sensitivity was more variable for ACE2 assays (0.17–0.93), with the lowest values observed for BA.2 Spike (0.42), BA.2 RBD (0.44), and BA.2.75 RBD (0.17).

### Pre-Omicron population antibody characteristics against SARS-CoV-2

SARS-CoV-2 ECL IgG concentrations generally increased with age and appeared to plateau around early adulthood across antigens (Fig 1A), although estimates at younger ages were imprecise because of the small number of participants, particularly among children aged <5 years. This pattern was consistent regardless of the specific antigen, indicating high cross-reactivity within the panel. Antibody concentrations in unvaccinated and uninfected participants remained consistently low compared to the other three categories across all variants and panels. Within each age group (<5 years, 5−17 years, 18 years and above), the GMC of IgG antibodies against SARS-CoV-2 Spike proteins showed substantial differences. For children under 5 years, GMCs ranged from 43 AU (95% CI: 5.4−333) [BA.2.75] to 78 AU (95% CI: 7.1−852) [Original strain]. For children aged 5–17 years, GMCs ranged from 626 AU (95% CI: 175−2,233) [BA.2.75] to 2,743 AU (95% CI: 629−11,958) [B.1.617.2]. And in adults older than 18 years, GMCs ranged from 115,117 AU (95% CI: 69,347−191,098) [B.1.351] to 192,086 AU (95% CI: 126,210−292,348) [Original strain]. PN assay results showed patterns similar to the ECL IgG results for the original SARS-CoV-2 strain and the BA.1 variant, with antibody responses increasing with age and reaching a plateau in early adulthood (Fig 1B).

**Fig 1:**
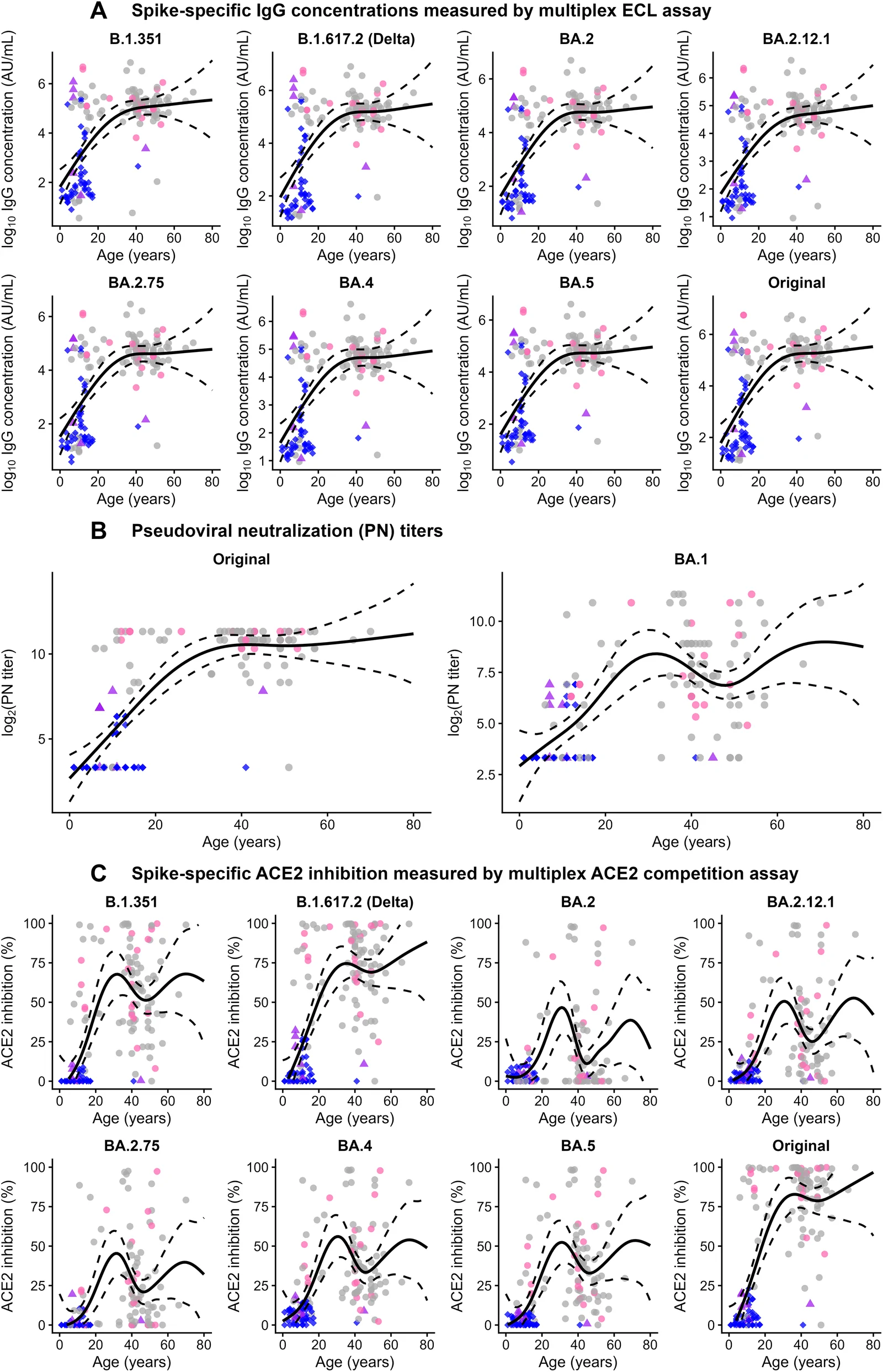
Age-specific SARS-CoV-2 antibody responses by assay type and immune status before Omicron emergence. (A) Spike-specific IgG concentrations measured by multiplex ECL assay. (B) PN titers against the original SARS-CoV-2 strain and BA.1 variant. (C) Spike-specific ACE2 inhibition measured by multiplex ACE2 competition assay. Points represent individual serum samples. Solid black lines indicate GAMM-fitted trends by age; dashed black lines indicate 95% confidence intervals. Colors indicate pre-Omicron immune status: hybrid immunity (pink), prior infection only (purple), vaccinated only (gray), and unvaccinated and uninfected (blue). ACE2, angiotensin-converting enzyme 2; ECL, electrochemiluminescence; GAMM, generalized additive mixed model; PN, pseudoviral neutralization.

A similar trend was observed for ACE2 assay results against the original SARS-CoV-2 strain, where percent inhibition increased with age and plateaued in early adulthood (Fig 1C). However, patterns varied among different antigens in adults. Percent inhibition was highest for the original strain (81.7%, 95% CI: 79.2%–84.3%), followed by higher values for the B.1.351 variant (58.5%, 95% CI: 55.2%–61.7%) and the Delta (B.1.617.2) variant (73.2%, 95% CI: 70.3%–76.0%). In contrast, percent inhibition was lower for other BA variants, with mean inhibition ranging from 22.7% for BA.2 to 41.5% for BA.4. Results in the prior infection alone group showed a lower percent inhibition, more similar to unvaccinated, uninfected participants across antigens, which differs from the patterns observed in ECL results. When comparing the original strain to other antigens, percent inhibition exhibited a wide range (0%–100%) in adults for non-original antigens. This finding suggests stronger cross-reactivity between the vaccine strain (original), B.1.351, and Delta variants, but less pronounced cross-reactivity between the original strain and later-emerging BA variants.

## Discussion

This study offers a comprehensive characterization of pre-Omicron antibody levels using multiple serological assessment tools in a longitudinal household cohort population with known COVID-19 vaccination and infection history. Across the cohort, we observed high antibody concentrations in vaccinated individuals and cross-reactivity across SARS-CoV-2 variants. Correlations between assays varied by exposure history and assay type, with electrochemiluminescence binding assays showing strong internal consistency and pseudovirus neutralization assays providing more accurate functional results.

We observed an age-related pattern in which pre-Omicron antibody levels generally increased through childhood and adolescence and appeared to plateau in early adulthood. This pattern should be interpreted cautiously because of the small numbers of younger participants and the wide confidence intervals around age-specific estimates. This dip most likely reflects timing rather than biology: adolescents and young adults often received vaccination closer to the serology draw, while many in their forties had a longer interval since the last vaccine or infection, resulting in lower recent antibody concentration. Second, the majority of our cohort participants are middle-aged with more heterogeneous exposure histories and product mixes, which can widen variance and pull smoothed estimates downward. Finally, the flexible spline in the model may reflect local fluctuations when sample sizes are small.

When stratifying by vaccination and infection history, correlations between antibody measurements were generally higher among vaccinated individuals. In contrast, correlation coefficients among previously infected individuals were more variable, particularly for ACE2 inhibition assays. Some BA.2 comparisons showed near-zero or slightly negative correlations when compared to original, delta, beta, and other omicron variants, which could result from either unique antigenic differences for BA.2, or greater uncertainty in assessing an antigenically drifted strain. Within each panel, antibody concentrations across different SARS-CoV-2 strains were highly correlated, reflecting high cross-reactivity. This pattern likely reflects recognition of conserved epitopes across variants or the presence of broadly binding but non-neutralizing antibodies [3].

We also assessed the nucleocapsid antibody results to identify potential asymptomatic infections not captured through PCR testing. Although the cohort conducted active surveillance for respiratory illness with systematic PCR testing of symptomatic illness, several participants who tested negative by PCR were seropositive for N, suggesting undetected prior infection. This finding indicates that even within a well-characterized cohort with active surveillance, some infections may not be captured through PCR-based case ascertainment, likely due to asymptomatic infections, mild illness that did not prompt swab collection, or infections occurring outside the surveillance period. N-based serology therefore provides complementary information for identifying prior infection history in longitudinal cohort studies. While N antibodies may wane over time, their presence remains a useful marker for differentiating infection-induced responses from vaccine-induced ones, especially in populations with low testing coverage or limited symptom reporting.

The strengths of this study include the use of multiple validated assay types, stratification by exposure status, and detailed evaluation of cross-variant immune responses. Limitations include the small overall sample size and particularly small numbers in some subgroups, including participants younger than 5 years and those with prior infection only, resulting in imprecise estimates for some age- and exposure-specific comparisons. However, the low antibody levels observed in some participants support the accurate classification of immune status. Future improvements to multiplex platforms, including enhanced variant-specific detection and reduced cross-reactivity, will further expand their value.

In conclusion, our results from this serosurvey nested within a prospective longitudinal household cohort provide important context for interpreting future cross-sectional population serosurveys, which lack detailed information on prior infection and vaccination history.

## Supporting information

S1 Fig Correlation of SARS-CoV-2 antibody measurements across assay platforms and variants. Heatmaps showing Spearman correlation coefficients between SARS-CoV-2 antibody measurements obtained using multiplex electrochemiluminescence (ECL) IgG assays, ACE2 inhibition assays, and pseudoviral neutralization (PN) assays. (A) All participants (N = 117). (B) Participants with prior SARS-CoV-2 infection only (N = 4). (C) Vaccinated-only participants (N = 71). Correlation coefficients range from −1.0 to 1.0. Darker shading indicates stronger correlations, and gray cells indicate negative correlations.
(DOCX)

S1 Table Diagnostic performance of multiplex ECL IgG and ACE2 inhibition assays for identifying prior COVID-19 vaccination among HIVE participants. AUC, optimal thresholds, sensitivity, and specificity for SARS-CoV-2 Spike and RBD antigens measured using ECL IgG and ACE2 inhibition assays.
(DOCX)
