# Supplementary material for: Evaluation of pre-Omicron population immunity for SARS-CoV-2 in the Household Influenza Vaccine Evaluation (HIVE) cohort
Source: PLoS One. 2026 Oct 15;21(10):e0360506. doi: 10.1371/journal.pone.0360506 (PMC13671514; doi:10.1371/journal.pone.0360506)
Supplement: Supplementary file 1 [file pone.0360506.s001.docx]

**Supplementary materials**

**Evaluation of pre-Omicron population immunity for SARS-CoV-2 in the Household Influenza Vaccine Evaluation (HIVE) cohort**

Authors: Yangyupei Yang^1^, Casey Juntila-Raymond^1^, Amy Callear^1^, Matthew Smith^1^, Reilly K. Atkinson^2^, Yan Zhao^2^, Ruhi Shah^2^, Jefferson J. S. Santos^2^, Scott E. Hensley^2^, Arnold S. Monto^1^, Emily T. Martin^1^

**Affiliation:**

^1^ Department of Epidemiology, University of Michigan School of Public Health, Ann Arbor, MI, USA.

^2^ Department of Microbiology, Perelman School of Medicine, University of Pennsylvania, Philadelphia, PA, USA.

**S1 Fig. Correlation of SARS-CoV-2 antibody measurements across assay platforms and variants.** Heatmaps showing Spearman correlation coefficients between SARS-CoV-2 antibody measurements obtained using multiplex electrochemiluminescence (ECL) IgG assays, ACE2 inhibition assays, and pseudoviral neutralization (PN) assays. Correlation coefficients range from −1.0 to 1.0. Darker shading indicates stronger correlations. Gray cells indicate negative correlations. Correlations were calculated using log10-transformed ECL IgG concentrations, percent ACE2 inhibition, and log2-transformed PN titers.

**(A) All participants (N = 117).**

**(B) Participants with prior SARS-CoV-2 infection only (N = 4).**

**(C) Vaccinated-only participants (N = 71).**
