# Supplementary material for: Evaluation of pre-Omicron population immunity for SARS-CoV-2 in the Household Influenza Vaccine Evaluation (HIVE) cohort
Source: PLoS One. 2026 Oct 15;21(10):e0360506. doi: 10.1371/journal.pone.0360506 (PMC13671514; doi:10.1371/journal.pone.0360506)
Supplement: Supplementary file 2 [file pone.0360506.s002.docx]

**Supplementary materials**

**Evaluation of pre-Omicron population immunity for SARS-CoV-2 in the Household Influenza Vaccine Evaluation (HIVE) cohort**

Authors: Yangyupei Yang^1^, Casey Juntila-Raymond^1^, Amy Callear^1^, Matthew Smith^1^, Reilly K. Atkinson^2^, Yan Zhao^2^, Ruhi Shah^2^, Jefferson J. S. Santos^2^, Scott E. Hensley^2^, Arnold S. Monto^1^, Emily T. Martin^1^

**Affiliation:**

^1^ Department of Epidemiology, University of Michigan School of Public Health, Ann Arbor, MI, USA.

^2^ Department of Microbiology, Perelman School of Medicine, University of Pennsylvania, Philadelphia, PA, USA.

**S1 Table. Diagnostic performance of multiplex ECL IgG and ACE2 inhibition assays for identifying prior COVID-19 vaccination among HIVE participants.**

| **Antigen** | **Assay panel^1^** | **AUC** | **Threshold^2^** | **Sensitivity (95% CI) ^3^** | **Specificity (95% CI)^3^** |
| --- | --- | --- | --- | --- | --- |
| SARS-CoV-2 Spike (B.1.351) | IgG Panel 29 | 0.84 | 4.3 | 0.89 (0.82-0.94) | 0.88 (0.75-0.95) |
| SARS-CoV-2 Spike (B.1.617.2; AY.4) Alt Seq 2 | IgG Panel 29 | 0.85 | 4.55 | 0.90 (0.82-0.95) | 0.87 (0.75-0.95) |
| SARS-CoV-2 Spike (BA.2) | IgG Panel 29 | 0.86 | 3.90 | 0.90 (0.83-0.95) | 0.88 (0.75-0.95) |
| SARS-CoV-2 Spike (BA.2.12.1) | IgG Panel 29 | 0.85 | 3.87 | 0.89 (0.82-0.94) | 0.88 (0.75-0.95) |
| SARS-CoV-2 Spike (BA.2.75) | IgG Panel 29 | 0.85 | 3.74 | 0.90 (0.83-0.95) | 0.87 (0.75-0.95) |
| SARS-CoV-2 Spike (BA.4) | IgG Panel 29 | 0.85 | 3.92 | 0.90 (0.83-0.95) | 0.88 (0.75-0.95) |
| SARS-CoV-2 Spike (BA.5) | IgG Panel 29 | 0.85 | 3.95 | 0.90 (0.83-0.95) | 0.88 (0.75-0.95) |
| SARS-CoV-2 Spike (Original) | IgG Panel 29 | 0.86 | 4.29 | 0.91 (0.84-0.96) | 0.88 (0.75-0.95) |
| RBD (B.1.1.7)(Alpha) | RBD Panel 30 | 0.86 | 3.89 | 0.90 (0.82-0.95) | 0.88 (0.75-0.95) |
| RBD (B.1.351)(Beta) | RBD Panel 30 | 0.86 | 3.48 | 0.90 (0.82-0.95) | 0.88 (0.75-0.95) |
| RBD (BA.2.12.1) | RBD Panel 30 | 0.86 | 3.32 | 0.90 (0.83-0.95) | 0.88 (0.75-0.95) |
| RBD (BA.2.75) | RBD Panel 30 | 0.88 | 3.20 | 0.89 (0.82-0.94) | 0.88 (0.75-0.95) |
| RBD (BA.4; BA.5) | RBD Panel 30 | 0.85 | 3.51 | 0.88 (0.80-0.93) | 0.88 (0.75-0.95) |
| RBD (L452R,T478K)(Delta) | RBD Panel 30 | 0.84 | 4.04 | 0.88 (0.80-0.93) | 0.88 (0.75-0.95) |
| RBD (Omicron; BA.2) | RBD Panel 30 | 0.87 | 3.43 | 0.90 (0.83-0.95) | 0.88 (0.75-0.95) |
| SARS-CoV-2 S1 RBD | RBD Panel 30 | 0.87 | 3.86 | 0.90 (0.83-0.95) | 0.88 (0.75-0.95) |
| SARS-CoV-2 Spike (Original) | ACE2 Panel 29 | 0.95 | 23.17 | 0.93 (0.87-0.97) | 1.00 (0.93-1.00) |
| SARS-CoV-2 Spike (B.1.351) | ACE2 Panel 29 | 0.96 | 12.08 | 0.90 (0.83-0.95) | 1.00 (0.93-1.00) |
| SARS-CoV-2 Spike (B.1.617.2; AY.4) Alt Seq 2 | ACE2 Panel 29 | 0.95 | 32.47 | 0.89 (0.82-0.94) | 1.00 (0.93-1.00) |
| SARS-CoV-2 Spike (BA.2) | ACE2 Panel 29 | 0.64 | 10.80 | 0.42 (0.33-0.52) | 0.98 (0.89-1.00) |
| SARS-CoV-2 Spike (BA.2.12.1) | ACE2 Panel 29 | 0.88 | 8.23 | 0.79 (0.71-0.86) | 0.94 (0.84-0.98) |
| SARS-CoV-2 Spike (BA.2.75) | ACE2 Panel 29 | 0.85 | 5.52 | 0.70 (0.61-0.79) | 0.94 (0.84-0.98) |
| SARS-CoV-2 Spike (BA.4) | ACE2 Panel 29 | 0.93 | 15.91 | 0.84 (0.76-0.90) | 0.98 (0.89-1.00) |
| SARS-CoV-2 Spike (BA.5) | ACE2 Panel 29 | 0.95 | 7.84 | 0.88 (0.81-0.93) | 0.98 (0.89-1.00) |
| RBD (B.1.1.7)(Alpha) | ACE2 Panel 30 | 0.95 | 22.28 | 0.90 (0.83-0.95) | 1.00 (0.94-1.00) |
| RBD (B.1.351)(Beta) | ACE2 Panel 30 | 0.94 | 15.15 | 0.88 (0.81-0.93) | 1.00 (0.93-1.00) |
| RBD (BA.2.12.1) | ACE2 Panel 30 | 0.88 | 15.77 | 0.78 (0.69-0.85) | 0.91 (0.80-0.97) |
| RBD (BA.2.75) | ACE2 Panel 30 | 0.53 | 29.31 | 0.17 (0.10-0.25) | 0.98 (0.91-1.00) |
| RBD (BA.4; BA.5) | ACE2 Panel 30 | 0.88 | 21.96 | 0.75 (0.66-0.82) | 0.96 (0.88-0.99) |
| RBD (L452R,T478K)(Delta) | ACE2 Panel 30 | 0.95 | 32.71 | 0.87 (0.80-0.93) | 1.00 (0.94-1.00) |
| RBD (Omicron; BA.2) | ACE2 Panel 30 | 0.69 | 22.95 | 0.44 (0.34-0.53) | 0.98 (0.91-1.00) |
| SARS-CoV-2 S1 RBD | ACE2 Panel 30 | 0.94 | 30.76 | 0.90 (0.83-0.95) | 1.00 (0.93-1.00) |

**Abbreviations:** AUC, area under the receiver operating characteristic curve; ACE2, angiotensin-converting enzyme 2; CI, confidence interval; ECL, electrochemiluminescence; IgG, immunoglobulin G.

^1^For IgG Panel 29 and RBD Panel 30, thresholds are reported as log10-transformed antibody concentrations (AU/mL). For ACE2 Panel 29 and ACE2 Panel 30 assays, thresholds are reported as percent inhibition (%).

^2^Optimal thresholds were determined using Youden's Index from receiver operating characteristic (ROC) curve analyses.

^3^Sensitivity and specificity were estimated using vaccination status as the reference classification and are presented with Wilson 95% confidence intervals.
