# Supplementary material for: Evaluation of pre-Omicron population immunity for SARS-CoV-2 in the Household Influenza Vaccine Evaluation (HIVE) cohort
Source: PLoS One. 2026 Oct 15;21(10):e0360506. doi: 10.1371/journal.pone.0360506 (PMC13671514; doi:10.1371/journal.pone.0360506)
Supplement: Supplementary file 3 [file pone.0360506.s003.docx]

**Evaluation of pre-Omicron population immunity for SARS-CoV-2 in the Household Influenza Vaccine Evaluation (HIVE) cohort**

**Manuscript Number:** PONE-D-26-31864

The authors present a nested serosurvey within the longitudinal HIVE household cohort, comparing ECL, ACE2 inhibition, and pseudovirus neutralization assays for pre-Omicron SARS-CoV-2 antibody characterization, and describing age-specific antibody trends using GAMM. This is a methodologically rich dataset: the pre-Omicron sampling window (July–December 2021) allows a genuinely clean separation between infection-induced and vaccine-induced immunity, which is difficult to achieve in most serosurveys conducted after Omicron's emergence. The three-way assay comparison (binding, ACE2-inhibition, and functional neutralization) is a valuable contribution in its own right, as is the use of N-protein serology to identify infections missed by active PCR surveillance. The detailed, prospectively collected vaccination and infection history available in this cohort is a genuine strength that most cross-sectional serosurveys lack. However, several internal inconsistencies in the reported sample/participant numbers and gaps in the statistical methods description should be resolved before the manuscript can be fully evaluated, as detailed below.

**Major Comments**

1. Results part, lines 178–184; The N-positivity figures in Section 3.3 do not reconcile: of 156 samples, 17 were N-positive, but only 6 with a prior PCR-confirmed infection and 9 without one are accounted for (15/17). These two categories should be mutually exclusive and exhaustive, so the gap suggests a data-linkage error or an undisclosed third category. Since this analysis supports the manuscript's claim that N-serology captures infections missed by PCR surveillance (Lines 264–271), please reconcile the denominator.

2. It is unclear whether the unit of analysis for the correlation and ROC analyses (Sections 3.2–3.4) is the participant (n=117) or the serum sample (n=156; Section 2.1, Lines 84–85, implies some participants contributed more than one sample). Please state explicitly whether repeated samples were treated as independent observations, since non-independence would affect the validity of the reported correlation coefficients, p-values, and AUC estimates. If so, please account for clustering (e.g., mixed-effects/GEE with a participant-level term) or restrict to one sample per participant.

3. The ROC analysis (Section 3.4, Lines 192–204) discriminates “vaccinated” from “unvaccinated” participants, but this grouping is not defined in the Statistical Analysis subsection (Section 2.4, Lines 137–152). Please specify which of the four immune-status categories (vaccinated only, hybrid immunity, prior infection only, unvaccinated/uninfected) fell on each side of this comparison, in particular how the hybrid-immunity and prior-infection-only groups were handled, since infection-induced antibodies in either group could materially affect the reported AUC, sensitivity, and specificity for distinguishing “vaccination status.”

4. Table 1 reports that “P values were calculated using Pearson's chi-square test” (Line 333) across all rows, including comparisons with very small or zero cell counts (e.g., Prior infection only, n=4; several zero cells in the Race row). Chi-square approximation is unreliable under these conditions; please indicate whether Fisher's exact test was used for sparse cells.

5. In Table 1, the Race and Ethnicity rows each sum to 116 rather than the stated overall N=117 (87+6+14+9=116; 112+4=116), and the Education row (restricted to adults) sums to 76 of 77 adults, with the “Vaccinated only” column summing to 60 rather than the 61 vaccinated-only adults reported in the text (Line 162). Please add a missing/unknown category or footnote to reconcile these totals.

6. Several age-stratified GMC estimates in Section 3.5 (Lines 212–216) come from very small subgroups (e.g., n=7 for the <5-years group) with correspondingly wide 95% CIs spanning roughly two orders of magnitude (e.g., 78 AU, 95% CI: 7.1–852). The conclusion that antibody levels “plateaued around 20 years of age” (Lines 207–208, 242–243) should acknowledge this imprecision rather than being stated without qualification.

**Minor Comments**

1. Section 2.3 (Line 110): “A result of 5000AU/mL N protein was considered seropositive” appears to be missing a comparison operator (≥ or >). Please state the exact cutoff rule.

2. Abstract (Lines 29–30), “Most were from adults and a majority received vaccination during the analysis period,” could be replaced with the actual figure given later in Results (97.4% of adults with vaccine-induced immunity, Line 161).

3. Figure 1B legend (Line 350) contains a stray period on its own line, apparently a formatting artifact.

4. Figure 1A axis label “log(IgG titer)” (Lines 344–346) is inconsistent with the AU/mL units used for GMCs in the text (e.g., Line 213); please align the axis label with the in-text units/terminology.

5. Section 2.4 (Lines 143–144) states "P values <0.05 were considered significant," but the correlation coefficients reported throughout Results (Sections 3.2–3.3, e.g., Lines 168–169, 186–188) are given only as ranges with a blanket "(p<0.05)" rather than exact values. Please report exact p-values, or at minimum the range of p-values, for the specific correlations discussed.

6. The summary sentence in Section 3.4 (Lines 202–204), "Overall, these findings indicate that IgG assays provided consistently strong discrimination of vaccination status, whereas ACE2 inhibition assays exhibited greater variant-specific heterogeneity despite maintaining high specificity," largely restates what is already reported earlier in the same section and again in the Discussion (Lines 238–241). Consider trimming one of these to reduce repetition.
