# Supplementary material for: Evaluation of pre-Omicron population immunity for SARS-CoV-2 in the Household Influenza Vaccine Evaluation (HIVE) cohort
Source: PLoS One. 2026 Oct 15;21(10):e0360506. doi: 10.1371/journal.pone.0360506 (PMC13671514; doi:10.1371/journal.pone.0360506)
Supplement: Supplementary file 4 [file pone.0360506.s005.docx]

**Evaluation of pre-Omicron population immunity for SARS-CoV-2 in the Household Influenza Vaccine Evaluation (HIVE) cohort**

**Manuscript Number:** PONE-D-26-31864

The authors present a nested serosurvey within the longitudinal HIVE household cohort, comparing ECL, ACE2 inhibition, and pseudovirus neutralization assays for pre-Omicron SARS-CoV-2 antibody characterization, and describing age-specific antibody trends using GAMM. This is a methodologically rich dataset: the pre-Omicron sampling window (July–December 2021) allows a genuinely clean separation between infection-induced and vaccine-induced immunity, which is difficult to achieve in most serosurveys conducted after Omicron's emergence. The three-way assay comparison (binding, ACE2-inhibition, and functional neutralization) is a valuable contribution in its own right, as is the use of N-protein serology to identify infections missed by active PCR surveillance. The detailed, prospectively collected vaccination and infection history available in this cohort is a genuine strength that most cross-sectional serosurveys lack. However, several internal inconsistencies in the reported sample/participant numbers and gaps in the statistical methods description should be resolved before the manuscript can be fully evaluated, as detailed below.

**Major Comments**

1. Results part, lines 178–184; The N-positivity figures in Section 3.3 do not reconcile: of 156 samples, 17 were N-positive, but only 6 with a prior PCR-confirmed infection and 9 without one are accounted for (15/17). These two categories should be mutually exclusive and exhaustive, so the gap suggests a data-linkage error or an undisclosed third category. Since this analysis supports the manuscript's claim that N-serology captures infections missed by PCR surveillance (Lines 264–271), please reconcile the denominator.

Thank you for identifying this inconsistency. We re-examined the linkage between N-serology results and prior PCR-confirmed infection history and reconciled all 17 N-positive samples. Specifically, 8 of the 17 N-positive samples were from participants with a documented PCR-confirmed infection before serum collection, and 9 were from participants without a documented prior PCR-confirmed infection (line 194-197).

2. It is unclear whether the unit of analysis for the correlation and ROC analyses (Sections 3.2–3.4) is the participant (n=117) or the serum sample (n=156; Section 2.1, Lines 84–85, implies some participants contributed more than one sample). Please state explicitly whether repeated samples were treated as independent observations, since non-independence would affect the validity of the reported correlation coefficients, p-values, and AUC estimates. If so, please account for clustering (e.g., mixed-effects/GEE with a participant-level term) or restrict to one sample per participant.

Thank you for requesting this clarification. The correlation and ROC analyses were conducted at the participant level rather than treating all 156 serum samples as independent observations. Specifically, one serum sample per participant was included in these analyses, resulting in 117 independent participant-level observations (line 148-151). We have now stated the unit of analysis and sample-selection procedure explicitly in method section and in the caption of supplemental figures.

3. The ROC analysis (Section 3.4, Lines 192–204) discriminates “vaccinated” from “unvaccinated” participants, but this grouping is not defined in the Statistical Analysis subsection (Section 2.4, Lines 137–152). Please specify which of the four immune-status categories (vaccinated only, hybrid immunity, prior infection only, unvaccinated/uninfected) fell on each side of this comparison, in particular how the hybrid-immunity and prior-infection-only groups were handled, since infection-induced antibodies in either group could materially affect the reported AUC, sensitivity, and specificity for distinguishing “vaccination status.”

Thank you. We agree that the vaccination-status classification used for the ROC analysis needed to be explicitly defined. We have revised to clarify that the vaccinated group included participants classified as vaccinated only or with hybrid immunity, whereas the unvaccinated group included participants classified as prior infection only or unvaccinated and uninfected (line 151-153). We have also clarified that these analyses evaluate discrimination of documented vaccination status in this cohort. Because prior infection can contribute to antibody responses independently of vaccination, we interpret these measures as cohort-specific discrimination of vaccination status rather than intrinsic diagnostic accuracy of the assays.

4. Table 1 reports that “P values were calculated using Pearson's chi-square test” (Line 333) across all rows, including comparisons with very small or zero cell counts (e.g., Prior infection only, n=4; several zero cells in the Race row). Chi-square approximation is unreliable under these conditions; please indicate whether Fisher's exact test was used for sparse cells.

We agree. Because several comparisons included small or zero cell counts, the chi-square approximation was not appropriate for all characteristics. We re-evaluated the Table 1 comparisons using Fisher's exact test for variables with sparse expected cell counts and Pearson's chi-square test where its assumptions were satisfied. We have updated the P values in Table 1 footnotes.

5. In Table 1, the Race and Ethnicity rows each sum to 116 rather than the stated overall N=117 (87+6+14+9=116; 112+4=116), and the Education row (restricted to adults) sums to 76 of 77 adults, with the “Vaccinated only” column summing to 60 rather than the 61 vaccinated-only adults reported in the text (Line 162). Please add a missing/unknown category or footnote to reconcile these totals.

Thank you for noting these discrepancies. The differences in totals reflect missing demographic information rather than inconsistencies in participant classification. Race and ethnicity information were each unavailable for one participant, and education information was unavailable for one adult participant. We have added this information to the Table 1 footnotes to make the variable-specific denominators clear. The vaccinated-only education total of 60 similarly reflects one missing education response among the 61 vaccinated-only adults.

6. Several age-stratified GMC estimates in Section 3.5 (Lines 212–216) come from very small subgroups (e.g., n=7 for the <5-years group) with correspondingly wide 95% CIs spanning roughly two orders of magnitude (e.g., 78 AU, 95% CI: 7.1–852). The conclusion that antibody levels “plateaued around 20 years of age” (Lines 207–208, 242–243) should acknowledge this imprecision rather than being stated without qualification.

We agree that the age-specific patterns should be interpreted more cautiously. We have revised the Abstract (35-37), Results (228-230), and Discussion (272-275, 306-309) to describe the observed pattern as an apparent plateau in early adulthood rather than a definitive age threshold. We also explicitly acknowledge the substantial uncertainty in younger age groups, particularly among children aged <5 years (n=7), as reflected by the wide confidence intervals around the GMC estimates. The limitations section has been revised accordingly (306-309).

**Minor Comments**

1. Section 2.3 (Line 110): “A result of 5000AU/mL N protein was considered seropositive” appears to be missing a comparison operator (≥ or >). Please state the exact cutoff rule.

≥ added in line 114

2. Abstract (Lines 29–30), “Most were from adults and a majority received vaccination during the analysis period,” could be replaced with the actual figure given later in Results (97.4% of adults with vaccine-induced immunity, Line 161).

Changes were made in line 28-29, 169-170

3. Figure 1B legend (Line 350) contains a stray period on its own line, apparently a formatting artifact.

The stray period was a formatting artifact and has been removed from the revised Figure 1 caption.

4. Figure 1A axis label “log(IgG titer)” (Lines 344–346) is inconsistent with the AU/mL units used for GMCs in the text (e.g., Line 213); please align the axis label with the in-text units/terminology.

The Figure 1 caption has been revised accordingly.

5. Section 2.4 (Lines 143–144) states "P values <0.05 were considered significant," but the correlation coefficients reported throughout Results (Sections 3.2–3.3, e.g., Lines 168–169, 186–188) are given only as ranges with a blanket "(p<0.05)" rather than exact values. Please report exact p-values, or at minimum the range of p-values, for the specific correlations discussed.

Thank you. We agree that the blanket statement “p<0.05” did not provide useful information regarding the individual correlations. Because the purpose of these analyses was to characterize the magnitude and pattern of agreement across antibody measurements rather than to test a null hypothesis of zero correlation, we have removed the blanket significance statements and now report the Spearman correlation coefficients (ρ) directly. We also removed the general statement that P values <0.05 were considered significant where it pertained to these descriptive correlation analyses.

6. The summary sentence in Section 3.4 (Lines 202–204), "Overall, these findings indicate that IgG assays provided consistently strong discrimination of vaccination status, whereas ACE2 inhibition assays exhibited greater variant-specific heterogeneity despite maintaining high specificity," largely restates what is already reported earlier in the same section and again in the Discussion (Lines 238–241). Consider trimming one of these to reduce repetition.

We agree and have removed the repetitive summary sentence from Section 3.4 to improve concision.
