# Supplementary material for: Evaluation of pre-Omicron population immunity for SARS-CoV-2 in the Household Influenza Vaccine Evaluation (HIVE) cohort
Source: PLoS One. 2026 Oct 15;21(10):e0360506. doi: 10.1371/journal.pone.0360506 (PMC13671514; doi:10.1371/journal.pone.0360506)
Supplement: Peer review history

# Peer review: Decision Letter 0

**DOI:** 10.1371/journal.pone.0360506.r001

**Authors:** Esaki M. Shankar

3 Sep 2026

Dear Dr. Yang,

plosone@plos.org. When you're ready to submit your revision, log on to https://www.editorialmanager.com/pone/ and select the 'Submissions Needing Revision' folder to locate your manuscript file.

- A letter that responds to each point raised by the academic editor and reviewer(s). You should upload this letter as a separate file labeled 'Response to Reviewers'.
- A marked-up copy of your manuscript that highlights changes made to the original version. You should upload this as a separate file labeled 'Revised Manuscript with Track Changes'.
- An unmarked version of your revised paper without tracked changes. You should upload this as a separate file labeled 'Manuscript'.

If you would like to make changes to your financial disclosure, please include your updated statement in your cover letter. Guidelines for resubmitting your figure files are available below the reviewer comments at the end of this letter.

If applicable, we recommend that you deposit your laboratory protocols in protocols.io to enhance the reproducibility of your results. Protocols.io assigns your protocol its own identifier (DOI) so that it can be cited independently in the future. For instructions see: https://journals.plos.org/plosone/s/submission-guidelines#loc-laboratory-protocols. Additionally, PLOS One offers an option for publishing peer-reviewed Lab Protocol articles, which describe protocols hosted on protocols.io. Read more information on sharing protocols at https://plos.org/protocols?utm_medium=editorial-email&utm_source=authorletters&utm_campaign=protocols.

As the corresponding author, your ORCID iD is verified in the submission system and will appear in the published article. PLOS supports the use of ORCID, and we encourage all coauthors to register for an ORCID iD and use it as well. Please encourage your coauthors to verify their ORCID iD within the submission system before final acceptance, as unverified ORCID iDs will not appear in the published article. *Only*  the individual author can complete the verification step; PLOS staff *cannot*  verify ORCID iDs on behalf of authors.

We look forward to receiving your revised manuscript.

Kind regards,

Esaki M. Shankar, PhD, FRCPath

Academic Editor

PLOS One

Journal Requirements:

When submitting your revision, we need you to address these additional requirements.

1.Please ensure that your manuscript meets PLOS ONE's style requirements, including those for file naming. The PLOS ONE style templates can be found at

https://journals.plos.org/plosone/s/file?id=wjVg/PLOSOne_formatting_sample_main_body.pdf and

https://journals.plos.org/plosone/s/file?id=ba62/PLOSOne_formatting_sample_title_authors_affiliations.pdf

2. Please note that PLOS One has specific guidelines on code sharing for submissions in which author-generated code underpins the findings in the manuscript. In these cases, we expect all author-generated code to be made available without restrictions upon publication of the work. Please review our guidelines at https://journals.plos.org/plosone/s/materials-and-software-sharing#loc-sharing-code and ensure that your code is shared in a way that follows best practice and facilitates reproducibility and reuse.

3. Please provide additional details regarding participant consent. In the ethics statement in the Methods and online submission information, please ensure that you have specified (1) whether consent was informed and (2) what type you obtained (for instance, written or verbal, and if verbal, how it was documented and witnessed). If your study included minors, state whether you obtained consent from parents or guardians. If the need for consent was waived by the ethics committee, please include this information. If you are reporting a retrospective study of medical records or archived samples, please ensure that you have discussed whether all data were fully anonymized before you accessed them and/or whether the IRB or ethics committee waived the requirement for informed consent. If patients provided informed written consent to have data from their medical records used in research, please include this information.

3. We note that the grant information you provided in the ‘Funding Information’ and ‘Financial Disclosure’ sections do not match.

When you resubmit, please ensure that you provide the correct grant numbers for the awards you received for your study in the ‘Funding Information’ section.

4. Thank you for stating the following financial disclosure:

“This project has been funded in whole with Federal funds from the National Institute of Allergy and Infectious Diseases, National Institutes of Health, Department of Health and Human Services, under Contract No. 75N93021C00015”

Please state what role the funders took in the study.  If the funders had no role, please state: "The funders had no role in study design, data collection and analysis, decision to publish, or preparation of the manuscript."

If this statement is not correct you must amend it as needed.

Please include this amended Role of Funder statement in your cover letter; we will change the online submission form on your behalf.

5. Thank you for stating the following in the Competing Interests section:

“We have no direct conflicts of interest to report related to this research. Outside of this work, A.S.M. has received CDC funding and grants from pharmaceutical consultations with Roche, Ltd. E.T.M. has received CDC funding and grants from pharmaceutical consultations with Merck and Co. S.E.H. is a co-inventor on patents that describe the use of nucleoside-modified mRNA as a vaccine platform. S.E.H reports receiving consulting fees from Sanofi, Pfizer, Lumen, Novavax, and Merck. S.E.H. and E.T.M. receive research funding from NIH UH2AI176136 awarded to Meso Scale Diagnostics, LLC, for unrelated work.”

We note that one or more of the authors are employed by a commercial company:”Roche, Ltd., Merck and Co., Sanofi, Pfizer, Lumen, Novavax, and Merck and NIH UH2AI176136 awarded to Meso Scale Diagnostics, LLC”

1. Please provide an amended Funding Statement declaring this commercial affiliation, as well as a statement regarding the Role of Funders in your study. If the funding organization did not play a role in the study design, data collection and analysis, decision to publish, or preparation of the manuscript and only provided financial support in the form of authors' salaries and/or research materials, please review your statements relating to the author contributions, and ensure you have specifically and accurately indicated the role(s) that these authors had in your study. You can update author roles in the Author Contributions section of the online submission form.

Please also include the following statement within your amended Funding Statement.

“The funder provided support in the form of salaries for authors “A.S.M., E.T.M, S.E.H ”, but did not have any additional role in the study design, data collection and analysis, decision to publish, or preparation of the manuscript. The specific roles of these authors are articulated in the ‘author contributions’ section.”

If your commercial affiliation did play a role in your study, please state and explain this role within your updated Funding Statement.

2. Please also provide an updated Competing Interests Statement declaring this commercial affiliation along with any other relevant declarations relating to employment, consultancy, patents, products in development, or marketed products, etc.

Within your Competing Interests Statement, please confirm that this commercial affiliation does not alter your adherence to all PLOS ONE policies on sharing data and materials by including the following statement: "This does not alter our adherence to  PLOS ONE policies on sharing data and materials.” (as detailed online in our guide for authors http://journals.plos.org/plosone/s/competing-interests) . If this adherence statement is not accurate and  there are restrictions on sharing of data and/or materials, please state these. Please note that we cannot proceed with consideration of your article until this information has been declared.

Please include both an updated Funding Statement and Competing Interests Statement in your cover letter. We will change the online submission form on your behalf.

6. Please note that funding information should not appear in any section or other areas of your manuscript. We will only publish funding information present in the Funding Statement section of the online submission form. Please remove any funding-related text from the manuscript.

7. Your ethics statement should only appear in the Methods section of your manuscript. If your ethics statement is written in any section besides the Methods, please move it to the Methods section and delete it from any other section. Please ensure that your ethics statement is included in your manuscript, as the ethics statement entered into the online submission form will not be published alongside your manuscript.

8. Please upload a new copy of Figure 1A, 1B and 1C as the detail is not clear. Please follow the link for more information:  https://journals.plos.org/plosone/s/figures.

9. Please include captions for your Supporting Information files at the end of your manuscript, and update any in-text citations to match accordingly. Please see our Supporting Information guidelines for more information: http://journals.plos.org/plosone/s/supporting-information.

10. If the reviewer comments include a recommendation to cite specific previously published works, please review and evaluate these publications to determine whether they are relevant and should be cited. There is no requirement to cite these works unless the editor has indicated otherwise.

Additional Editor Comments:

The authors should carefully address the concerns on the statistical analyses raised by the reviewer before submitting a revision.

[Note: HTML markup is below. Please do not edit.]

Reviewer's Responses to Questions

**Comments to the Author**

1. Is the manuscript technically sound, and do the data support the conclusions?

Reviewer #1: Yes

**********

2. Has the statistical analysis been performed appropriately and rigorously?

Reviewer #1: Yes

**********

3. Have the authors made all data underlying the findings in their manuscript fully available?

The PLOS Data policy

Reviewer #1: Yes

**********

4. Is the manuscript presented in an intelligible fashion and written in standard English?

Reviewer #1: Yes

**********

Reviewer #1: Evaluation of pre-Omicron population immunity for SARS-CoV-2 in the Household Influenza Vaccine Evaluation (HIVE) cohort

Manuscript Number: PONE-D-26-31864

The authors present a nested serosurvey within the longitudinal HIVE household cohort, comparing ECL, ACE2 inhibition, and pseudovirus neutralization assays for pre-Omicron SARS-CoV-2 antibody characterization, and describing age-specific antibody trends using GAMM. This is a methodologically rich dataset: the pre-Omicron sampling window (July–December 2021) allows a genuinely clean separation between infection-induced and vaccine-induced immunity, which is difficult to achieve in most serosurveys conducted after Omicron's emergence. The three-way assay comparison (binding, ACE2-inhibition, and functional neutralization) is a valuable contribution in its own right, as is the use of N-protein serology to identify infections missed by active PCR surveillance. The detailed, prospectively collected vaccination and infection history available in this cohort is a genuine strength that most cross-sectional serosurveys lack. However, several internal inconsistencies in the reported sample/participant numbers and gaps in the statistical methods description should be resolved before the manuscript can be fully evaluated, as detailed below.

Major Comments

1. Results part, lines 178–184; The N-positivity figures in Section 3.3 do not reconcile: of 156 samples, 17 were N-positive, but only 6 with a prior PCR-confirmed infection and 9 without one are accounted for (15/17). These two categories should be mutually exclusive and exhaustive, so the gap suggests a data-linkage error or an undisclosed third category. Since this analysis supports the manuscript's claim that N-serology captures infections missed by PCR surveillance (Lines 264–271), please reconcile the denominator.

2. It is unclear whether the unit of analysis for the correlation and ROC analyses (Sections 3.2–3.4) is the participant (n=117) or the serum sample (n=156; Section 2.1, Lines 84–85, implies some participants contributed more than one sample). Please state explicitly whether repeated samples were treated as independent observations, since non-independence would affect the validity of the reported correlation coefficients, p-values, and AUC estimates. If so, please account for clustering (e.g., mixed-effects/GEE with a participant-level term) or restrict to one sample per participant.

3. The ROC analysis (Section 3.4, Lines 192–204) discriminates “vaccinated” from “unvaccinated” participants, but this grouping is not defined in the Statistical Analysis subsection (Section 2.4, Lines 137–152). Please specify which of the four immune-status categories (vaccinated only, hybrid immunity, prior infection only, unvaccinated/uninfected) fell on each side of this comparison, in particular how the hybrid-immunity and prior-infection-only groups were handled, since infection-induced antibodies in either group could materially affect the reported AUC, sensitivity, and specificity for distinguishing “vaccination status.”

4. Table 1 reports that “P values were calculated using Pearson's chi-square test” (Line 333) across all rows, including comparisons with very small or zero cell counts (e.g., Prior infection only, n=4; several zero cells in the Race row). Chi-square approximation is unreliable under these conditions; please indicate whether Fisher's exact test was used for sparse cells.

5. In Table 1, the Race and Ethnicity rows each sum to 116 rather than the stated overall N=117 (87+6+14+9=116; 112+4=116), and the Education row (restricted to adults) sums to 76 of 77 adults, with the “Vaccinated only” column summing to 60 rather than the 61 vaccinated-only adults reported in the text (Line 162). Please add a missing/unknown category or footnote to reconcile these totals.

Minor Comments

1. Section 2.3 (Line 110): “A result of 5000AU/mL N protein was considered seropositive” appears to be missing a comparison operator (≥ or >). Please state the exact cutoff rule.

2. Abstract (Lines 29–30), “Most were from adults and a majority received vaccination during the analysis period,” could be replaced with the actual figure given later in Results (97.4% of adults with vaccine-induced immunity, Line 161).

3. Figure 1B legend (Line 350) contains a stray period on its own line, apparently a formatting artifact.

4. Figure 1A axis label “log(IgG titer)” (Lines 344–346) is inconsistent with the AU/mL units used for GMCs in the text (e.g., Line 213); please align the axis label with the in-text units/terminology.

5. Section 2.4 (Lines 143–144) states "P values <0.05 were considered significant," but the correlation coefficients reported throughout Results (Sections 3.2–3.3, e.g., Lines 168–169, 186–188) are given only as ranges with a blanket "(p<0.05)" rather than exact values. Please report exact p-values, or at minimum the range of p-values, for the specific correlations discussed.

**********

what does this mean?). If published, this will include your full peer review and any attached files.

If you choose “no”, your identity will remain anonymous but your review may still be made public.

**Do you want your identity to be public for this peer review?**  For information about this choice, including consent withdrawal, please see our Privacy Policy

Reviewer #1: No

**********

[NOTE: If reviewer comments were submitted as an attachment file, they will be attached to this email and accessible via the submission site. Please log into your account, locate the manuscript record, and check for the action link "View Attachments". If this link does not appear, there are no attachment files.]

To ensure your figures meet our technical requirements, please review our figure guidelines: https://journals.plos.org/plosone/s/figures

You may also use PLOS’s free figure tool, NAAS, to help you prepare publication quality figures: https://journals.plos.org/plosone/s/figures#loc-tools-for-figure-preparation.

NAAS will assess whether your figures meet our technical requirements by comparing each figure against our figure specifications.

Attachment Submitted filename: Comments.docx

# Author comment: Author response to Decision Letter 1

**DOI:** 10.1371/journal.pone.0360506.r002

8 Sep 2026

Major Comments

1. Results part, lines 178–184; The N-positivity figures in Section 3.3 do not reconcile: of 156 samples, 17 were N-positive, but only 6 with a prior PCR-confirmed infection and 9 without one are accounted for (15/17). These two categories should be mutually exclusive and exhaustive, so the gap suggests a data-linkage error or an undisclosed third category. Since this analysis supports the manuscript's claim that N-serology captures infections missed by PCR surveillance (Lines 264–271), please reconcile the denominator.

Thank you for identifying this inconsistency. We re-examined the linkage between N-serology results and prior PCR-confirmed infection history and reconciled all 17 N-positive samples. Specifically, 8 of the 17 N-positive samples were from participants with a documented PCR-confirmed infection before serum collection, and 9 were from participants without a documented prior PCR-confirmed infection (line 194-197).

2. It is unclear whether the unit of analysis for the correlation and ROC analyses (Sections 3.2–3.4) is the participant (n=117) or the serum sample (n=156; Section 2.1, Lines 84–85, implies some participants contributed more than one sample). Please state explicitly whether repeated samples were treated as independent observations, since non-independence would affect the validity of the reported correlation coefficients, p-values, and AUC estimates. If so, please account for clustering (e.g., mixed-effects/GEE with a participant-level term) or restrict to one sample per participant.

Thank you for requesting this clarification. The correlation and ROC analyses were conducted at the participant level rather than treating all 156 serum samples as independent observations. Specifically, one serum sample per participant was included in these analyses, resulting in 117 independent participant-level observations (line 148-151). We have now stated the unit of analysis and sample-selection procedure explicitly in method section and in the caption of supplemental figures.

3. The ROC analysis (Section 3.4, Lines 192–204) discriminates “vaccinated” from “unvaccinated” participants, but this grouping is not defined in the Statistical Analysis subsection (Section 2.4, Lines 137–152). Please specify which of the four immune-status categories (vaccinated only, hybrid immunity, prior infection only, unvaccinated/uninfected) fell on each side of this comparison, in particular how the hybrid-immunity and prior-infection-only groups were handled, since infection-induced antibodies in either group could materially affect the reported AUC, sensitivity, and specificity for distinguishing “vaccination status.”

Thank you. We agree that the vaccination-status classification used for the ROC analysis needed to be explicitly defined. We have revised to clarify that the vaccinated group included participants classified as vaccinated only or with hybrid immunity, whereas the unvaccinated group included participants classified as prior infection only or unvaccinated and uninfected (line 151-153). We have also clarified that these analyses evaluate discrimination of documented vaccination status in this cohort. Because prior infection can contribute to antibody responses independently of vaccination, we interpret these measures as cohort-specific discrimination of vaccination status rather than intrinsic diagnostic accuracy of the assays.

4. Table 1 reports that “P values were calculated using Pearson's chi-square test” (Line 333) across all rows, including comparisons with very small or zero cell counts (e.g., Prior infection only, n=4; several zero cells in the Race row). Chi-square approximation is unreliable under these conditions; please indicate whether Fisher's exact test was used for sparse cells.

We agree. Because several comparisons included small or zero cell counts, the chi-square approximation was not appropriate for all characteristics. We re-evaluated the Table 1 comparisons using Fisher's exact test for variables with sparse expected cell counts and Pearson's chi-square test where its assumptions were satisfied. We have updated the P values in Table 1 footnotes.

5. In Table 1, the Race and Ethnicity rows each sum to 116 rather than the stated overall N=117 (87+6+14+9=116; 112+4=116), and the Education row (restricted to adults) sums to 76 of 77 adults, with the “Vaccinated only” column summing to 60 rather than the 61 vaccinated-only adults reported in the text (Line 162). Please add a missing/unknown category or footnote to reconcile these totals.

Thank you for noting these discrepancies. The differences in totals reflect missing demographic information rather than inconsistencies in participant classification. Race and ethnicity information were each unavailable for one participant, and education information was unavailable for one adult participant. We have added this information to the Table 1 footnotes to make the variable-specific denominators clear. The vaccinated-only education total of 60 similarly reflects one missing education response among the 61 vaccinated-only adults.

6. Several age-stratified GMC estimates in Section 3.5 (Lines 212–216) come from very small subgroups (e.g., n=7 for the <5-years group) with correspondingly wide 95% CIs spanning roughly two orders of magnitude (e.g., 78 AU, 95% CI: 7.1–852). The conclusion that antibody levels “plateaued around 20 years of age” (Lines 207–208, 242–243) should acknowledge this imprecision rather than being stated without qualification.

We agree that the age-specific patterns should be interpreted more cautiously. We have revised the Abstract (35-37), Results (228-230), and Discussion (272-275, 306-309) to describe the observed pattern as an apparent plateau in early adulthood rather than a definitive age threshold. We also explicitly acknowledge the substantial uncertainty in younger age groups, particularly among children aged <5 years (n=7), as reflected by the wide confidence intervals around the GMC estimates. The limitations section has been revised accordingly (306-309).

Minor Comments

1. Section 2.3 (Line 110): “A result of 5000AU/mL N protein was considered seropositive” appears to be missing a comparison operator (≥ or >). Please state the exact cutoff rule.

≥ added in line 114

2. Abstract (Lines 29–30), “Most were from adults and a majority received vaccination during the analysis period,” could be replaced with the actual figure given later in Results (97.4% of adults with vaccine-induced immunity, Line 161).

Changes were made in line 28-29, 169-170

3. Figure 1B legend (Line 350) contains a stray period on its own line, apparently a formatting artifact.

The stray period was a formatting artifact and has been removed from the revised Figure 1 caption.

4. Figure 1A axis label “log(IgG titer)” (Lines 344–346) is inconsistent with the AU/mL units used for GMCs in the text (e.g., Line 213); please align the axis label with the in-text units/terminology.

The Figure 1 caption has been revised accordingly.

5. Section 2.4 (Lines 143–144) states "P values <0.05 were considered significant," but the correlation coefficients reported throughout Results (Sections 3.2–3.3, e.g., Lines 168–169, 186–188) are given only as ranges with a blanket "(p<0.05)" rather than exact values. Please report exact p-values, or at minimum the range of p-values, for the specific correlations discussed.

Thank you. We agree that the blanket statement “p<0.05” did not provide useful information regarding the individual correlations. Because the purpose of these analyses was to characterize the magnitude and pattern of agreement across antibody measurements rather than to test a null hypothesis of zero correlation, we have removed the blanket significance statements and now report the Spearman correlation coefficients (ρ) directly. We also removed the general statement that P values <0.05 were considered significant where it pertained to these descriptive correlation analyses.

6. The summary sentence in Section 3.4 (Lines 202–204), "Overall, these findings indicate that IgG assays provided consistently strong discrimination of vaccination status, whereas ACE2 inhibition assays exhibited greater variant-specific heterogeneity despite maintaining high specificity," largely restates what is already reported earlier in the same section and again in the Discussion (Lines 238–241). Consider trimming one of these to reduce repetition.

We agree and have removed the repetitive summary sentence from Section 3.4 to improve concision.

Attachment Submitted filename: response to reviwers comments.docx

# Peer review: Decision Letter 1

**DOI:** 10.1371/journal.pone.0360506.r003

**Authors:** Esaki M. Shankar

29 Sep 2026

Evaluation of pre-Omicron population immunity for SARS-CoV-2 in the Household Influenza Vaccine Evaluation (HIVE) cohort

PONE-D-26-31864R1

Dear Dr. Yang,

We’re pleased to inform you that your manuscript has been judged scientifically suitable for publication and will be formally accepted for publication once it meets all outstanding technical requirements.

Within one week, you’ll receive an e-mail detailing the required amendments. When these have been addressed, you’ll receive a formal acceptance letter and your manuscript will be scheduled for publication.

An invoice will be generated when your article is formally accepted. Please note, if your institution has a publishing partnership with PLOS and your article meets the relevant criteria, all or part of your publication costs will be covered. Please make sure your user information is up-to-date by logging into Editorial Manager at Editorial Manager® and clicking the ‘Update My Information' link at the top of the page. For questions related to billing, please contact billing support.

If your institution or institutions have a press office, please notify them about your upcoming paper to help maximize its impact. If they’ll be preparing press materials, please inform our press team as soon as possible -- no later than 48 hours after receiving the formal acceptance. Your manuscript will remain under strict press embargo until 2 pm Eastern Time on the date of publication. For more information, please contact onepress@plos.org.

Kind regards,

Esaki M. Shankar, PhD, FRCPath

Academic Editor

PLOS One

Additional Editor Comments (optional):

Reviewers' comments:

Reviewer's Responses to Questions

**Comments to the Author**

Reviewer #1: All comments have been addressed

**********

2. Is the manuscript technically sound, and do the data support the conclusions?

Reviewer #1: Yes

**********

3. Has the statistical analysis been performed appropriately and rigorously?

Reviewer #1: Yes

**********

4. Have the authors made all data underlying the findings in their manuscript fully available?

The PLOS Data policy

Reviewer #1: Yes

**********

5. Is the manuscript presented in an intelligible fashion and written in standard English?

Reviewer #1: Yes

**********

Reviewer #1: The authors have satisfactorily addressed all of my previous comments. The reconciliation of the N-positivity data, the clarification of the unit of analysis and the vaccination status grouping for the ROC analysis, and the more cautious interpretation of the age-specific antibody patterns have improved the clarity and rigor of the manuscript. I have no further concerns and recommend acceptance.

**********

what does this mean?). If published, this will include your full peer review and any attached files.

If you choose “no”, your identity will remain anonymous but your review may still be made public.

**Do you want your identity to be public for this peer review?**  For information about this choice, including consent withdrawal, please see our Privacy Policy

Reviewer #1: No

**********

# Editor report: Acceptance letter

**DOI:** 10.1371/journal.pone.0360506.r004

**Authors:** Esaki M. Shankar

PONE-D-26-31864R1

PLOS One

Dear Dr. Yang,

I'm pleased to inform you that your manuscript has been deemed suitable for publication in PLOS One. Congratulations! Your manuscript is now being handed over to our production team.

At this stage, our production department will prepare your paper for publication. This includes ensuring the following:

* All references, tables, and figures are properly cited

* All relevant supporting information is included in the manuscript submission,

* There are no issues that prevent the paper from being properly typeset

You will receive further instructions from the production team, including instructions on how to review your proof when it is ready. Please keep in mind that we are working through a large volume of accepted articles, so please give us a few days to review your paper and let you know the next and final steps.

Lastly, if your institution or institutions have a press office, please let them know about your upcoming paper now to help maximize its impact. If they'll be preparing press materials, please inform our press team within the next 48 hours. Your manuscript will remain under strict press embargo until 2 pm Eastern Time on the date of publication. For more information, please contact onepress@plos.org.

You will receive an invoice from PLOS for your publication fee after your manuscript has reached the completed accept phase. If you receive an email requesting payment before acceptance or for any other service, this may be a phishing scheme. Learn how to identify phishing emails and protect your accounts at https://explore.plos.org/phishing.

If we can help with anything else, please email us at customercare@plos.org.

Thank you for submitting your work to PLOS One and supporting open access.

Kind regards,

PLOS One Editorial Office Staff

on behalf of

Dr. Esaki M. Shankar

Academic Editor

PLOS One
